# Training university teachers and students in Sri Lanka on Gender Based Violence: testing of a participatory training program

**DOI:** 10.15694/mep.2018.0000043.1

**Published:** 2018-02-22

**Authors:** Pia Axemo, Kumudu Wijewardena, Ruvani Fonseka, Sharika Cooray, Elisabeth Darj

**Affiliations:** 1 Women´s and Children`s Health/IMCH; 2 Department of Community Medicine Health; 3 Joint Doctoral Program in Public Health; 4 United Nations Population Fund; 5 Department of Public Health and Nursing

**Keywords:** training program, gender based violence, gender, universities, Sri Lanka

## Abstract

This article was migrated. The article was marked as recommended.

In all societies, violence is a social problem and violation of human rights. Changing attitudes and behaviors, which accept violence at individual and societal levels are key components in prevention strategies.

The aim of this study was to produce educational material on Gender Based Violence (GBV). A participatory study design including educators and university students was used to create four teaching modules. The teaching was evaluated by descriptive surveys before and after the training and focus group discussions followed the training session. The questionnaire covered attitudes to gender, violence and laws. One hundred eleven teachers and 25 students representing different faculties and universities participated in separate workshops in three Sri Lankan universities. The students lacked knowledge of the meaning of GBV, consequences and existing laws. Women held more gender-equitable attitudes. Both women and men favoured equal participation of work and decision in the households. Male undergraduates showed less accepting attitudes toward rape or blaming women for rape Three categories emerged after the FGDs; Make training module compulsory and teacher led; Mind your own business; What can be done.

The newly prepared and context specific material was well-received by educators and students and they provided valuable inputs, which improved the educational modules.

## Introduction

In all societies, globally and at the local level, violence against women and men is a social problem, as well as a violation of human rights. Changing attitudes and behavior that permit violence at the individual and societal levels is a key component in violence prevention strategies.

Gender based violence (GBV) is defined by WHO as; “Any act of gender-based violence that results in, or is likely to result in, physical, sexual or psychological harm or suffering to women, including threats of such acts, coercion or arbitrary deprivations of liberty, whether occurring in public or in private life.” ( World Health Organization 2013).

The definition clearly outlines the nature of GBV, however it must be noted that violence cannot be confined only to women. While men and boys also experience GBV, the limited evidence suggests that it is not to the same extent as to women. A WHO multi-country study conducted in 2013 indicated that one in three women are victims of physical/sexual violence in their lifetime, which is evidence of the pervasive nature and extent of GBV ( World Health Organization 2013). GBV has wide-ranging implications for health and well-being, such as loss of confidence or self-worth, psychosocial trauma, suicide and homicide.

In Sri Lanka, many segments of society have been made vulnerable by the recent three-decade civil conflict, exposing people to higher risk of GBV. Additionally, the country’s militarization and the impunity enjoyed by perpetrators have aggravated the situation causing the normalization of a culture of violence ( De Mel, Peiris and Gomez cited 2013).

Although studies on GBV in Sri Lanka are limited, a survey covering 11 districts in 2011 showed that 51.2% of the respondents, both men and women, reported experience of domestic violence ( De Mel et.al. 2013). A more recent study performed by UNFPA indicated that 90% of women are affected by sexual harassment on public transport (UNFPA 2017), and a Sri Lankan study among 1322 undergraduates, 41% male and 58% female, mean age 22 years, indicated that 44% have faced sexual abuse, while 36% have faced physical abuse ( Fernando and Karunasekere 2011). Another study in 2013 highlighted that one in three ever-partnered men, reported having committed physical and/or sexual violence against an intimate partner in their lifetime, 20% reported having committed sexual violence against an intimate partner and 28% of the men had themselves experienced sexual violence ( De Mel et al.2013).

South East Asia has the highest rate of GBV, according to WHO (WHO), however due to weak Sri Lankan national data the full magnitude of the issue is not known. The 2016 Demographic and Health Survey highlighted that 17 % of women interviewed had suffered any form of domestic violence the last 12 months ( Department of Census and Statistics 2017). Sri Lankan universities have been reported as sites for sexual and GBV ( Straus 2004; Preventing Sexual and Gender based Violence Strategies for Universities 2013). In spite of having a rich culture and heritage of imparting knowledge in education, universities in Sri Lanka have had an increasing culture of “ragging,” harassment of underclassmen which is both physical and sexual in nature (Ragging at Universities 2017; Lekamwasam et al. 2015; Hennayake 2008 *).*

Ragging has been highlighted as a shortcoming of the national universities, hindering a quality educational experience for students who entered the system with great difficulty, as only 16 % of students eligible to apply for university studies are accepted every year at the 15 government universities (Statistical Bulletin on Education). In spite of several policies aiming to improve this situation, GBV is still ongoing in the university sector. In a meta-analysis from 21 countries, the relationship between violence in childhood and educational outcomes demonstrated that, child maltreatment of all forms increased the risk of bad educational outcomes, poorer working memory performance and more mental health problems ( Fry et al. 2017; Romano, Babchishin, Marquis and Frechette 2015; Trank,van Berkel,van Jzendoorn and Alink 2017). There have been many reported cases where students have dropped out or had their academic performance suffer because of maltreatment ( Fry et al.2017; Fernando,Miller and Berger 2010). To guarantee high quality education, a culture of security is necessary within the universities, based on values of dignity and respect and with zero tolerance of GBV. Having identified this need for more awareness on preventing GBV within national universities, there is a knowledge gap to be filled. In a recent qualitative study on attitudes related to GBV among male university students in Colombo, those young men strongly suggested including education regarding GBV early in their study curriculum, at least at the university level if not before ( Darj, Wijewardena, Lindmark and Axemo 2017).

The aim of this study was to produce training material on GBV in collaboration with United Nations Population Fund (UNFPA) and a university in Colombo, Sri Lanka, with input from educators and students at different universities in Sri Lanka, in order to increase knowledge and awareness about gender attitudes and patriarchal norms which result in violence.

## Methods

### Study design

A participatory study design was used to create the training material. This study design was employed to plan and conduct the study with the community members whose actions were under study ( Bergold & Thomas 2012).

The training material was evaluated by a descriptive quantitative analysis and focus group discussions (FGDs) following a pilot training session among the students. Further, an open group discussion followed each presentation of the training material among educators.

### Study setting

The first training sessions, for both educators and students, took place at a university in Colombo, and six months later in two other universities outside the capital: one in the Northern province and another one in the Eastern province.

### Study participants

Fifty educators, with equal number of men and women, representing five different universities, both from the capital and from different provinces attended pilot presentations of the training material, consisting of four modules. In a separate pilot training session, 25 students, 9 men and 16 women, from one university in Colombo attended. They were selected to get a broad representation from different faculties (Applied Sciences, Humanities and Social Sciences, and Management Studies) and representing both first year and more senior (third and fourth year) students. These students attended all four educational modules and were afterwards invited to voluntarily participate in the subsequent FGDs. Eight men and seven female students accepted the invitation and were included.

After six months, two more workshops took place: one in the Northern and one in the Eastern Province. In one university 32 and in the other 29 middle level academic staff participated. In both universities staff members from disciplines of Humanities, Management, Agriculture, Applied Sciences, Medicine, and Communication voluntarily participated due to their interest in teaching the topic GBV to students and other staff members.

### Study tools

The training material aimed to achieve the following outcomes: provide a deeper understanding on the issues of gender, sex, and violence; how to address and prevent GBV; and was developed through different phases.

A team was formed among researchers and activists from one university in Colombo, Uppsala University, Sweden and UNFPA Sri Lanka to develop the first training material composed of four different modules:

Module 1: differences between sex and gender

Module 2: various forms of GBV and its health and non-health impacts

Module 3: understanding contributing factors and the legal framework to GBV in Sri Lanka

Module 4: evidence-based ways of preventing and/or minimizing GBV

All modules included local and international statistics on GBV, as well as exercises to measure knowledge.

In order to test the training materials, collaborators from a Colombo university, local UNFPA (at the first training session) and Uppsala University held four interactive workshops, three for educators and one for students. During and following the training sessions, an open discussion was encouraged through trainee-led group-works, case studies, and facilitator-led discussions. The first workshop with educators proceeded for four hours. The student workshop lasted 4.5 hours and the remaining two educator workshops took 1.5 days each. The final training material was then produced taking into consideration all inputs from the educators and students.

### Data collection

The educators answered and discussed 12 gender attitude questions at the beginning of the session. The students answered a pre- and post-training questionnaire, a shorter version of the CARE questionnaire ( De Mel et al. 2013), validated in Sri Lanka including the same 12 attitude questions given to the educators. The questionnaire contained questions related to age, relationship status, attitudes to gender and violence, and knowledge of the legal climate in relation to GBV. The post-test included the same questions.

Two FGDs, with a duration of one hour each, followed the training, with male and female students in separate groups. FGD was considered suitable for investigating the students’ perceptions on GBV in their own community, utilizing group interactions. A topic guide for the FGD was prepared in advance by the researchers. The guide covered the students’ perceptions of the teaching, the content of the teaching material, harassment climate at the universities, and actions to be taken to reduce GBV. The FGDs were held in English with explanations in Sinhalese, if needed. FGDs were tape recorded with permission from the participants and took place in two separate conference halls at the university.

### Data analysis

#### Quantitative

The students’ responses to the pre- and post-training questionnaires were analyzed using Microsoft Excel for Mac, version 15. The analysis was descriptive, comparing pre- and post- answers to each question among the 16 women and 9 men who completed surveys before and after the training session. In all attitude questions, students were provided four answer options: “strongly disagree,” “disagree,” “agree,” and “strongly agree.” For the purposes of comparison, these four answer options were collapsed into the binary answers of “disagree” or “agree” in the analysis.

#### Qualitative

Qualitative content analysis of the FGDs was performed by text condensation ( Graneheim & Lundman 2004). The transcripts were read and re-read several times by PA, KW and ED and units of meaning were identified and condensed. The condensed units were coded and merged into subcategories and further into categories.

### Ethical consideration

All participants gave their consent to participate prior to the training, and were aware that no names were recorded or would be mentioned and the material would be keep in a locked place reachable to only one of the authors. Due to the sensitivity of the topic covered, a medical doctor/counselor was appointed in case any of the participants needed consultation following the workshop. However, this service was not utilized by the participants. The research team obtained ethical clearance from the SIDCER (Strategic Initiative for Developing Capacity in Ethical Review) recognized Ethical Review Committee (ERC) in the Faculty of Medical Sciences, University of Sri Jayewardenepura Gangodawila, Nugegoda, Sri Lanka (REF 59/14).

## Results

### Quantitative results

The educators assessed the attitude questions during their training session and found them relevant and possible for university students to understand and answer. In a discussion with the researchers, some of the teachers proposed that the training modules should include more about violence against men, with more national and local statistics and examples of national gender norms. They also suggested including more exercises, role-plays and case studies and extending the training time to one full day.

One of the key findings among the students was the lack of knowledge and understanding of the meaning of gender-based violence and its implications/consequences. All 25 students were between ages 20-24 years, and none had been married, but half of them had a boy- or girlfriend. The undergraduates were asked questions to reveal their attitudes about gender ( Table 1) Women undergraduates generally held more gender-equitable attitudes answering the pre-teaching questionnaire than men. In the pre-test, a majority of the women consider a womens´ most important role is to take care of the household, and after the training less than half still held that opinion. Both men and women thought that taking part in household work was a role for both partners. However, both disagreed with the statement ‘Changing nappies, giving kids a bath and feeding the kid is a mothers responsibility’ a statement which only 1/16 women and 3/9 men agreed with in the posttest. Almost all of the men and women did not agree, neither before nor after the training, to the statement ‘A women cannot refuse to have sex with her husband’. Only one woman and two men agreed to the statement in the post-questionnaire ( Table 1).

**Table 1.** Attitudes of students pre and post training (those who selected ‘agree’ or ‘strongly agree’)

Question	Women (n = 16)		Men (n =9)	
	Pre	Post	Pre	Post
A woman’s most important role is to take care of her home and cook for her family	11	7	4	3
There are times when a woman deserves to be beaten	1	1	1	1
To be a man, you need to be tough	2	2	4	1
Changing nappies, giving kids a bath and feeding the kids are the mother’s responsibility	3	1	2	3
To be a man means providing for your family and your extended family	6	6	5	3
It is manly to defend the honour of your family even by violent means	2	3	4	4
A woman should obey her husband	2	8	5	7
A man should have the final say in all family matters	1	0	4	2
Men should share the work around the house with women such as doing dishes, cleaning, and cooking	14	14	8	8
A woman cannot refuse to have sex with her husband	1	1	2	2
When a woman is raped, she is usually to blame for putting herself in that situation	2	0	3	0
If a woman doesn’t physically fight back, it’s not rape	7	1	3	0
In any rape case, one would have to question whether the victim is promiscuous or has a bad reputation	6	3	4	2
Some women ask to be raped by the way they dress and behave	9	9	7	8
I support greater participation of women in coming forward for elections	10	13	8	7
I think more women should be in public decision making roles	10	14	8	8

The undergraduate men who participated showed positive changes in several attitudes, particularly with regard to rape. In particular, after the workshop, no male students agreed with the following two statements: ‘When a woman is raped, she is usually to blame for putting herself in that situation,’ and ‘If a woman doesn’t physically fight back, it’s not rape’, For the women students, there was a slight change after the training towards less agreement with the two above statements. However, a majority of the men and women showed agreement to the question ‘Some women ask to be raped the way they dress or behave’, with women showing no change from pre- to post-test while for men 7/9 agreed before and 8/9 after training. There were also changes in some attitudes away from gender equity; after the workshop, almost all of the men agreed with the statement ‘A woman should obey her husband’, and half of the women agreed. A majority of all both at pre-and posttest thought that women should come forward in elections and public decisions.

Finally, the questionnaires asked the students about laws in Sri Lanka regarding GBV and their opinions and the opinions of their communities on the topic. Very few answers changed greatly from pre- to post-questionnaire. One change was that nearly half of men and women undergraduates answered ‘don’t know’ before the training but majority answered ‘yes’ after the training to ‘According to the law, is a husband who forces his wife to have sex against her will committing a criminal act’ ( Table 2). Around half of both men (4/9) and women (8/16) found the questionnaire easy to answer.

**Table 2.** Law questions

Questions	Women (n = 16)		Men (n =9)	
	Pre	Post	Pre	Post
According to the law, is a husband who forces his wife to have sex against her will committing a criminal act (that is, the husband can be fined or put in jail)?				
Yes	5	10	3	6
No	1	3	1	1
Don’t Know	7	3	4	2
Are there any laws in your country about violence against women?				
Yes	13	16	9	8
No	0	0	0	1
Don’t Know	0	0	0	0

### Findings from focus group discussions

After the analyses of the data three main categories emerged with eight subcategories.

**Table:** 

Category	Subcategories
Make training module compulsory and teacher led	• *Importance of dedicated educator to lead compulsory education on GBV* • *Bi- or tri- lingual education* • *Gender difference in the interest of GBV*
“Mind your own business”	• *Strong social norms, people do not act* • *Violence at the university*
What can be done	• Tasks of the University • Students unions don´t work • Wishes for the future

### Make training modules compulsory and teacher led

Overall, the participants found the training modules appropriate and needed, as they had not received any such training before during their educational experiences.

Most of the students don´t have any knowledge about those facts [GBV]. (Women’s FGD, abbreviated; FGDW).

Even if it is good to introduce the training at university level. It should have started earlier - you should have sessions for schoolchildren. They are ignorant about such things (FGDW).

Most of the students’ knowledge on matters related to GBV came from the media. They believed that the presentation of violence in the media was incorrect and exaggerated, and that the role of the media should be to give nonjudgmental information on GBV.

To have the modules internet-based for individual education was discussed during the FGDs. However, students expressed the wish to make the modules educator-led and not internet-based, but stressed.

There must be dedicated teachers. (Men’s FGD abbreviated FGDM).

The presentations were done in English as that was the language of instruction for the students in their university programs. However, in the discussions it became evident that both the female and male groups would like the material to be available bi- or even tri-lingual:

When you are explaining using both languages [English and Sinhalese] we can understand each and everything, but then you using [our] mother tongue definitely it touches our hearts (FGDM).

They also recommended that both men and women should participate in the same group during training and that the presentation also included harassment against boys.

When you say GBV what comes to mind is always only ladies (FGDW).

The students agreed that generally women and girls show more interest in the topic of GBV, but if the vulnerable situation of young men at campus also is discussed that would generate more interest from male participants. Some of the discussions during the training could also be in separate groups, as the women felt that they expressed themselves less in a mixed-gender group. Pedagogically, the students asked for more real-life case stories, especially examples matching their own student life situation. They also suggested more pictures and perhaps a video, and if time permits, more discussions. They recommended that the training be presented when students arrive in the university and have their compulsory orientations in their introductory weeks.

### Mind your own business

When discussing whether anyone had witnessed or heard of violence or sexual harassment in the public or private sphere, most of the participants replied affirmatively. They commonly referred to sexual harassment in public transportations.

In public transportation, no one will speak up..maybe talk later in person (FGDW).

At the universities, both men and women were aware of the harsh situation for first year students (‘freshers’), especially those coming from rural areas and having no local networks.

Because if a boy gets abused by another..most of the fresher´s face that experience, but the matter is they have nowhere to go (FGDM).

At least they don´t talk to parents and seniors will suppress [them] (FGDM).

The students gave examples of both physical and mental violence that mostly happened to these rural students. People coming from the Colombo area are protected by their preexisting networks.

My school is in Colombo, so this is my area, so I am never afraid, that´s my force (FGDM).

The female students had also noticed that male students could suffer violence if they helped a girl but that boy didn´t take any action.

A boy that was escorting me to the gate at around 8 pm after studying, was hit in front of me, they[senior male students] thought he kept me [didn´t let me go]. I was shocked, he didn´t want to [tell] because he said it will cause problems between the faculties. He was a boy from a village, he just kept quiet, he told me not to tell others (FGDW).

Several of the students felt that the norm in the society was: ‘To look away’, a mentality has grown to;

Mind your own business kind of attitude and there are others who judge you for talking about others’ problems (FGDW).

The female students reaffirmed, as well as the male students that they also understand and notice that men are harassed at campuses, but nobody talks about it, not students nor teachers.

When you say GBV what comes to mind is always ladies (FGDW).

They also felt that the lecturers as well as the university administration ignored the occurring violence.

We have nobody to turn to (FGDM).

Their joint conclusion was that the violent behaviour will be repeated by the current freshers to younger students when they become senior students.

### What can be done?

The discussions moved from the university level to the individual level. In order to gain support from educators the students felt that the educators need training on GBV. The students also need possibilities to contact trusted staff to whom they could reveal personal experiences. The contact could be face to face or internet-based if anonymity could be secured. As ragging drew a lot of attention, the young men suggested increased supervision such as surveillance cameras, safety guards or other solutions.

As I told you there are many secret officers [guards] in the government and we can employ them in the university premises. (FGDM).

There are button cameras (FGDM).

Even though the students felt that educators looked away when ragging occured on campus, they still proposed that it would be:

Very good to have a society with senior lecturers or mature people to have a small group that look into students´ situation (FGDM).

Though every faculty has student unions, they were not considered a viable option as they are run only by men and are highly politicized, with ongoing fights and women are not welcomed.

Voting is not open, not all students vote, some selected people vote. We don´t mind boys being in the unions as long as they do the right things. If we can get them take the message [about GBV/ragging] it would be great and more effective (FGDW).

As individuals, the boys proposed discussion fora/clubs to discuss GBV and other relevant youth topics. Some proposed a system where senior students act as mentors for the freshers and introduce and support them in their new university life. In the female FGD, one women with support from others discussed boys’ and girls’ different upbringing, with different rights and responsibilities. But she believed her generation could be the change.

I have a brother and I get angry when I see a difference. I just tell - wait and see how I raise my son (FGDW).

Contrary to the male FGD, the women reflected over the cultural norms of being a man and a woman in their own society and violence in general.

Men have more power than women. Men are given more (FGDW).

In our country, in the Asian culture, it´s like ok for a man to be violent (FGDW).

Even the mothers say like the girls should give in..it is ok for a boy to be violent (FGDW).

In order to decrease violence, they agreed.

We all must learn to RESPECT each other irrespective of gender or sex (FGDW).

## Discussion

The four modules were appreciated and evoked interest. Some minor changes need to be done, such as more information on violence against boys and men, more group discussion and case studies. It is important to have compulsory training with dedicated teachers and for the training to come early when entering the university. Participants also wanted the training to be ongoing and linked to other advocacy activities. Students need trusted persons on campus to contact if suffering violence or witnessing violence. This is very much in line with results from a study on dating violence and sexual assault at American universities ( Chiari, Verdiglione and Zadik 2017). The students asked for more training among educators and students, and for trusted educators and counsellors to consult. They also suggested a greater focus on underserved groups, such as men suffering sexual violence.

In the FGD, women students focused on the men exerting power and control, but did not discuss any changes that women or girls needed to make in their own gender attitudes. The women though mentioned that they would change the upbringing of boys when they start their own families. In an earlier qualitative study in Sri Lanka among young male academics, they expressed a view that women should stand up against gender discrimination and develop strong personalities. But the academics did not mention in which way they themselves could change their “male” personalities ( Darj et.al 2017).

Neither the women nor the men discussed if men should change their gender attitudes or roles. The same attitudes were reflected in a set of studies from Glasgow ( McCarrie &Lombard 2016) of 11-12 years old school children and then 10 years later a group of 15-18 year olds and their understanding of men´s violence against women. In both studies, the children normalized violence. The boys fail to acknowledge their own role in unequal gender norms, and both girls and boys had internalized patriarchal social norms. The same tendency can be observed in our study from 2014 ( Darj et al. 2017) and also in this study where the gender norms are quite consolidated, even if both men and women seems to be willing to take a more active role when it comes to household work and upbringing of children.

We also found that the students accept heteronormativity. One example was the way they discuss clothes. Women’s clothing are being sexualized and women are defined by how men view them, also demonstrated in earlier studies ( De Mel et al. 2013; Darj et al. 2017).

The knowledge about laws in Sri Lanka around GBV was low among students. After the training there was a misunderstanding among the students that marital rape is a criminal act despite it being explicitly exempted from criminality under Sri Lankan laws None of the students had heard about the anti-ragging law that has existed since 1998. That means that the students are entering the university world without knowing anything about, for example, codes of conduct or the legal system that could have serious implications in their lives. These knowledge gaps also became evident in a study from US university campuses ( Chiari et al. 2017).

University students are in a formative period in their lives, especially in relation to development of appropriate patterns of behavior toward a partner. Universities have a duty and opportunity to be inclusive and work against discrimination regarding languages, ethnicities, religion, gender, urban/rural origin. In order to reach these goals, universities should consider basic training for both staff and students on GBV, as it is a serious problem both physically and mentally in most parts of the world. Also establishing the effect violence has on the learning capacities of a person merits further attention. When constructing the training material, it should be accessible to all and in a country like Sri Lanka with three main language, it must be trilingual, in Sinhalese, Tamil and English ( Davis 2015).

The fact that victims of violence often first approach peers or trusted educators in a university setting makes education on GBV, and how to act as responder or bystander, both for students and university staff, very important ( Chiari et al. 2017).

When students after a harsh selection, where only 16 % of those applying to universities are selected, enter this new world, they may feel considerable stress and anxiety but also satisfaction (Statistical Bulletin on Education). They will unfortunately encounter what is called ragging or bullying. Ragging is mentioned in the media, but few studies have been done. One from the Faculty of Dentistry in Sri Lanka demonstrated that 50% of the students have experienced any form of mistreatment and 18 % experienced sexual harassment ( Premadasa, Wanigasooriya, Thalib and Ellepole 2011). The students in the FGD mentioned ragging and that it was a problem that was overlooked in the campuses. A smaller intervention was tried in one medical university in the south of the country.

The staff identified key stakeholders that could have an influence on the students’ behavior and attitudes. The group included senior students, academics, alumni of the university, clinical teachers, a selected group of parents of the new students and security officers. The group defined together ragging in operational terms and how it should be reported. Strict rules for interaction of senior and junior students were decided and the staff had to carefully supervise and report. Regular contact with parents of the newcomers was established. The observation period was just during the two introductory weeks and no incidents were noticed during this period. The study shows a model how an anti-ragging group could work ( Lekamwasam et al. 2015). Another article in Asian Tribune consider ragging as a fundamental threat to Sri Lankan universities and demonstrate how the senior students and student unions are mainly responsible ( Hennayake 2008). We also noticed that the students are not well-informed about the laws related to ragging or other forms of violence. If negative ways of interacting are not stopped, such patterns of interaction can persist when the young people continue their adult life after graduation. Studies related to young university people and violence against dating partners in 16 countries (five in Asia), indicated that there is a strong correlation between cultural norms of violence acceptance in a society and interpersonal behavior, an important factor that need to be considered and discussed at universities ( Straus 2004). University staff and students need to discuss what codes of conduct should apply for both teachers and students and that the rules of law are valid at campus and at the university.

The intent of the training was to increase awareness of all sorts of violence, not just physical violence, but also the psychological and the impact that witnessing violence has on children. Moreover, it was also intended to develop an understanding that violence is unacceptable and to hopefully slowly change behavior. Even though the students were aware of the unequal power roles in the society, they had quite fixed ideas about what is the accepted behavior when it comes to gender roles. The female students reflected over their roles as future mothers and how to up bring up their children, but that discussion did not appear in the male students group. GBV do not refer to women only as the male students thought but the young men must also be encouraged to stand up for equality and equity ( Darj et al.2017; McCarrie and Lombard 2016). The female students stressed the need for men and women to jointly work towards a respectful behavior towards each other.

A UN multi-country study on men and violence in Asia and Pacific demonstrated that most first non-partner rape was perpetrated when male perpetrators were between 15-19 years of age, including in Sri Lanka ( De Mel et al. 2013). This means that in order to prevent violence, interventions must focus on childhood and adolescence to address culturally-rooted masculinity patterns ( De Koker P.Mathews C,Zuch M,Bastien S,Mason-Jones AJ 2013). The students who participated in the FGDs in this study as well as the study from 2014 and another among medical students in Sri Lanka clearly indicate that education should start early at home and in school and be continued at university level ( Straus 2004; Darj et.al 2017; Haj-Yahia & De Zousa 2007).

Straus et al, in their dating violence study also stressed that more than 57% of the students in the 16 countries had suffered corporal punishment as children and it was demonstrated that such treatment as a child would increase antisocial behavior and acceptance of violence with future partners and family members ( Straus 2004).

All educational programs and awareness training on GBV for students should have a solid understanding of gender and gender inequalities and also a component on how to develop healthy relationships.

## Strengths and Limitations

### Strengths

The joint development of the training material with persons from different settings including a government university, a big international organization working with questions related to GBV and researchers from two Nordic universities experienced in research and teaching on GBV from different continents was a major asset to the project. Additionally, the participatory development design collected inputs from a large group of teacher staff with different degrees of academic merits and made the teaching material context-specific with a local flavor known to all participants.

The author KW is a Co-chairperson of the Gender Equity and Equality Standing Committee of the Universities Grant Commission (UGC) which is planning and coordinating university education in Sri Lanka. The standing committee is the standard setter, policy maker, arbitrator of UGC policy on gender equity and equality in Sri Lankan universities. In most universities in Sri Lanka, there are Gender Cells, which can take this training forward with involvement from the UGC. Sri Lankan universities are also taking measures to stop violence due to ragging. Hopefully these modules, being introduced to new entrants and 3 ^rd^ year students of the universities, can lead to a change in their attitudes and behavior in the future.

The presentation of the modules was done jointly by the team in English, but also explained in Sinhala. The moderator (KW) in the male FGD is experienced in qualitative research as well as the observer (PA), with experiences in qualitative and quantitative research as well as research on violence in Sri Lanka. The moderator in the female group (SC) is experienced in moderating FGDs and the observers (ED) and (RF) both have experiences in research on violence and qualitative and quantitative research in multiple countries including Sri Lanka. University educators from different and widespread universities were able to give their input on the training in an open discussion. In order to get the input from the students we applied different methods such as small group discussions, case studies, questionnaires, and FGDs.

### Limitations

The sample of student was a small convenience sample. The views expressed only apply to these students and cannot be generalized even if their views are similar to those found in other studies from the country ( De Mel et al. 2013; Darj et al 2017; Premadasa et al. 2011; Haj-Yahia & De Zousa 2007). The methodology was non-experimental. We did not have a longitudinal follow up.

The post-test could only be done immediately after the training session, due to practical reasons to get the same students to come again weeks later, as a university student strike was ongoing. The time to answer all the questionnaires was limited and language problems could have contributed to low response rate on some questions.

The training had to be done in a rather short time, as the students had to participate in other compulsory activities, but as the developed training must be in a restricted time frame to accommodate university student schedules, this way of teaching reflected the way it will be conducted in the future.

## Conclusion

A newly prepared and context specific training material for university students was well-received by both university students and teachers. Both groups provided valuable input on how to make the material even more context-specific and accessible to all students in the form of a compulsory course during the universities’ introduction weeks.

With increased knowledge related to GBV at the campuses, students could be enabled to be non-judgmental supporters of peers who have experienced GBV, and to also consider respectful ways of interaction in future interpersonal relationships. Further research should have a longitudinal approach and also survey both educators’ and students’ experiences of violence, ragging and the received and given training, to better understand how to change university climates to prevent GBV.

## Take Home Messages

• Students lack knowledge on the meaning of GBV the consequences and existing laws
• Women held more gender-equitable attitudes
• Both men and women favored equal participation of work and decision at home
• GBV training modules appreciated and preferably to be compulsory introductory training material at universities

## Notes On Contributors

Pia Axemo is associate professor in International Maternal and Child Health and an obstetrician /gynecologist at the University of Uppsala, Sweden. She has a longstanding research collaboration with the University of Sri Jayewardenepura in Colombo, Sri Lanka and with Muhimbili University in Dar es Salaam, Tanzania.

Kumudu Wijewardena is professor in Community Health at Sri Jayewardenepura University in Colombo, Sri Lanka and a Co-chairperson of the Gender Equity and Equality Standing Committee of the Universities Grant Commission (UGC) which is planning and coordinating university education in Sri Lanka.

Ruvani Fonseka is a Harvard educated gender equity and global health Master of Public Health and Master of Social Welfare and has experiences of working in USA, Mali, India and Sri Lanka.

Sharika Cooray is a programe and policy analyst with focus on questions related to women´s rights and gender at the UNFPA regional office in Colombo, Sri Lanka.

Elisabeth Darj is professor at the Department of Public Health and Nursing at the Norwegian University of Science and Technology in Trondheim(NTNU), Norway. She started the first Master program in Global Health at NTNU She is also an obstetrician /gynecologist affiliated to the Universities of Uppsala and Trondheim.
