# Supplementary material for: Training university teachers and students in Sri Lanka on Gender Based Violence: testing of a participatory training program
Source: MedEdPublish (2016). 2018 Feb 22;7:43. [Version 1] doi: 10.15694/mep.2018.0000043.1 (PMC10711944; doi:10.15694/mep.2018.0000043.1)
Supplement: Peer review history

# Reviewer report: Reviewer response for version 1

**DOI:** 10.21956/mep.19517.r28879

**Published:** 2018-03-25

**Authors:** Janusz Janczukowicz

**Affiliations:** 1 Medical University of Lodz

This review has been migrated. The reviewer awarded 4 stars out of 5 While this paper concerns general higher education, it focuses on the extremely important issue of training both students and faculty in addressing and preventing gender-based violence. Definitely, the described training materials should be further developed and their educational effectiveness should be evaluated at possibly high levels of the Kirkpatrick model. I would suggest focusing further research on integration of particular GBV-training with whole curricula and institutional culture to avoid possible tensions between explicit teaching and hidden curriculum.

Reviewer Expertise:

NA

No decision status is available

# Reviewer report: Reviewer response for version 1

**DOI:** 10.21956/mep.19517.r28877

**Published:** 2018-03-01

**Authors:** Petra Verdonk

**Affiliations:** 1 VU University Medical Center, dpt Medical Humanities

This review has been migrated. The reviewer awarded 4 stars out of 5 The authors address an extremely important topic, which is often hardly integrated in medical education. In the introduction, the authors show that they understand the contextual nature of Gender Based Violence (GBV) within Sri Lankan society, a society in which violence against women is highly accepted. The authors issue the class-gender intersection in their description of ‘ragging’ against first generation students, which was a new and horrifying phenomenon to me. Teaching about these issues in medical education may be challenging especially in a violence-tolerant environment. Across the globe, medical educator must step up and introduce this issue in medical curricula given ts prevalence, its health consequences, and its societal consequences. Unfortunately, medical education itself is not free from this phenomenon, as many studies have also exposed medicine as a field in which sexual harassment is highly prevalent. The #MeToo movement shows how timely this issue still is in the workplace, and that includes the medical workforce.

Reviewer Expertise:

NA

No decision status is available

# Reviewer report: Reviewer response for version 1

**DOI:** 10.21956/mep.19517.r28878

**Published:** 2018-02-28

**Authors:** P Ravi Shankar

**Affiliations:** 1 American International Medical University

This review has been migrated. The reviewer awarded 4 stars out of 5 I enjoyed reading this manuscript about developing a module on gender based violence (GBV) in Sri Lanka. The authors have described the process adequately and proper ethical approval was obtained for the study. I obtained information about GBV in the Sri Lankan society. I have a question regarding why separate focus group discussions were conducted for male and female students. A broad range of teachers and students from various disciplines were included while developing and obtaining feedback about the GBV module. The quality of written English in the manuscript is good. Take home messages and the conclusions are based on the data presented during the course of the study. This paper will be of wide interest to educators and students in a variety of settings.

Reviewer Expertise:

NA

No decision status is available
